# O-GalNAc glycosylation determines intracellular trafficking of APP and Aβ production

**DOI:** 10.1016/j.jbc.2023.104905

**Published:** 2023-06-09

**Authors:** Yuriko Tachida, Junko Iijima, Kazuto Takahashi, Hideaki Suzuki, Yasuhiko Kizuka, Yoshiki Yamaguchi, Katsunori Tanaka, Miyako Nakano, Daisuke Takakura, Nana Kawasaki, Yuko Saito, Hiroshi Manya, Tamao Endo, Shinobu Kitazume

**Affiliations:** 1 Disease Glycomics Team, RIKEN, Wako, Saitama, Japan; 2 Department of Clinical Laboratory Sciences, School of Health Sciences, Fukushima Medical University School of Medicine, Fukushima, Japan; 3 Graduate School of Natural Science and Technology, Gifu University, Gifu, Japan; 4 Institute for Glyco-core Research (iGCORE), Gifu University, Gifu, Japan; 5 Division of Pharmaceutical Physical Chemistry, Tohoku Medical and Pharmaceutical University, Miyagi, Japan; 6 Department of Chemical Science and Engineering, School of Materials and Chemical Technology, Tokyo Institute of Technology, Meguro-ku, Tokyo, Japan; 7 Biofunctional Synthetic Chemistry Laboratory, RIKEN Cluster for Pioneering Research, Wako, Saitama, Japan; 8 Graduate School of Integrated Sciences for Life, Hiroshima University, Higashi-hiroshima, Japan; 9 Graduate School of Medical Life Science, Yokohama City University, Yokohama, Japan; 10 Department of Neuropathology, Tokyo Metropolitan Geriatric Hospital and Institute of Gerontology, Tokyo, Japan; 11 Molecular Glycobiology, Research Team for Mechanism of Aging, Tokyo Metropolitan Geriatric Hospital and Institute of Gerontology, Tokyo, Japan

**Keywords:** Alzheimer’s disease (AD), amyloid precursor protein (APP), endothelial cell, intracellular trafficking, O-glycosylation

## Abstract

A primary pathology of Alzheimer’s disease (AD) is amyloid β (Aβ) deposition in brain parenchyma and blood vessels, the latter being called cerebral amyloid angiopathy (CAA). Parenchymal amyloid plaques presumably originate from neuronal Aβ precursor protein (APP). Although vascular amyloid deposits’ origins remain unclear, endothelial APP expression in APP knock-in mice was recently shown to expand CAA pathology, highlighting endothelial APP’s importance. Furthermore, two types of endothelial APP—highly O-glycosylated APP and hypo-O-glycosylated APP—have been biochemically identified, but only the former is cleaved for Aβ production, indicating the critical relationship between APP O-glycosylation and processing. Here, we analyzed APP glycosylation and its intracellular trafficking in neurons and endothelial cells. Although protein glycosylation is generally believed to precede cell surface trafficking, which was true for neuronal APP, we unexpectedly observed that hypo-O-glycosylated APP is externalized to the endothelial cell surface and transported back to the Golgi apparatus, where it then acquires additional O-glycans. Knockdown of genes encoding enzymes initiating APP O-glycosylation significantly reduced Aβ production, suggesting this non-classical glycosylation pathway contributes to CAA pathology and is a novel therapeutic target.

Alzheimer’s disease (AD) is a progressive neurodegenerative disorder that features two pathological hallmarks: intraneuronal neurofibrillary tangles (1) and extracellular deposition of amyloid β (Aβ) (2). Aβ is generated from amyloid precursor protein (APP). When APP is cleaved at the plasma membrane at the α-site within the Aβ sequence, N-terminal ectodomain, sAPPα, is released, and subsequent γ-secretase cleavage of the carboxy-terminal fragment generates p3 peptide instead of Aβ (3). While part of cell surface APP is internalized and transported to the endosome, BACE1 protease cleaves at the β-site during the endocytic pathway (4), leading to shedding of the N-terminal ectodomain, sAPPβ, and subsequent cleavage of the carboxy-terminal fragment at the γ-site to produce Aβ. Therefore, the cellular level of Aβ production largely depends on the extent to which APP encounters each active secretase in the cell (5).

Both APP and its secretase are glycosylated, and several reports suggest that such glycosylation affects Aβ production. Unusual GalNAc-type O-glycosylation to a Tyr residue within the Aβ sequence, which is frequently found in cerebrospinal fluid from patients with AD (6), results in conformational changes in APP favorable for the amyloidogenic pathway (7). Modification of the N-glycans of BACE1 with bisecting GlcNAc attenuates its lysosomal targeting and enhances Aβ production (8), indicating that glycosylation can affect the intracellular localization of secretases to modulate Aβ production. Normally APP has two N- glycans at specific Asn residues and multiple GalNAc-type O-glycans at Ser/Thr residues (9, 10, 11, 12). N-glycosylation is initiated in the ER to generate oligomannose-type glycan; then, a series of Golgi-resident glycosidases and glycosyltransferases functions in the processing of N-glycans and the addition of GalNAc-type O-glycans (13), which are considered to be maturation steps of glycoproteins and necessary for their trafficking to functional locations. Notably, O-GalNAc glycoproteome analysis revealed that remarkable numbers of Golgi- and ER-resident proteins have O-glycans (9).

Aβ plaques are not limited to brain parenchyma, and vascular Aβ deposition, known as cerebral amyloid angiopathy (CAA), is also observed at a high frequency (14). Parenchymal Aβ is considered to originate from neuronal APP, but a recent finding showing that endothelial APP expression (15) contributes to vascular Aβ deposition (16) highlighted the importance of endothelial APP for the pathogenesis of CAA. Cell-type-specific mRNA splicing produces different APP isoforms in humans, namely, APP695, APP751, and APP770 (17). Neurons express APP695, whereas vascular endothelial cells (ECs) express APP770 (12), and APP751 shows a relatively ubiquitous expression pattern. Compared with APP695, APP751 has a KPI domain, and APP770 has KPI and OX2 domains.

In this study, we focused on the glycosylation and intracellular trafficking of neuronal APP695 and endothelial APP770. Contrary to neuronal APP695, in which both N- and O-linked glycans are attached to the APP before its cell surface transport, we found that endothelial APP770 takes a non-classical biosynthetic pathway; hypo-O-glycosylated APP, but having N-glycans, is transported to the cell surface and is then internalized and transported back to the Golgi apparatus for O-glycosylation. Our study sheds light on an overlooked functional connection between cell-type-dependent protein glycosylation and intracellular trafficking and also raises the possibility that modulation of the O-glycosylation pathway could attenuate cellular Aβ production.

## Results

### O-glycosylated sAPP is shed from neurons and endothelial cells

**Figure 1:**
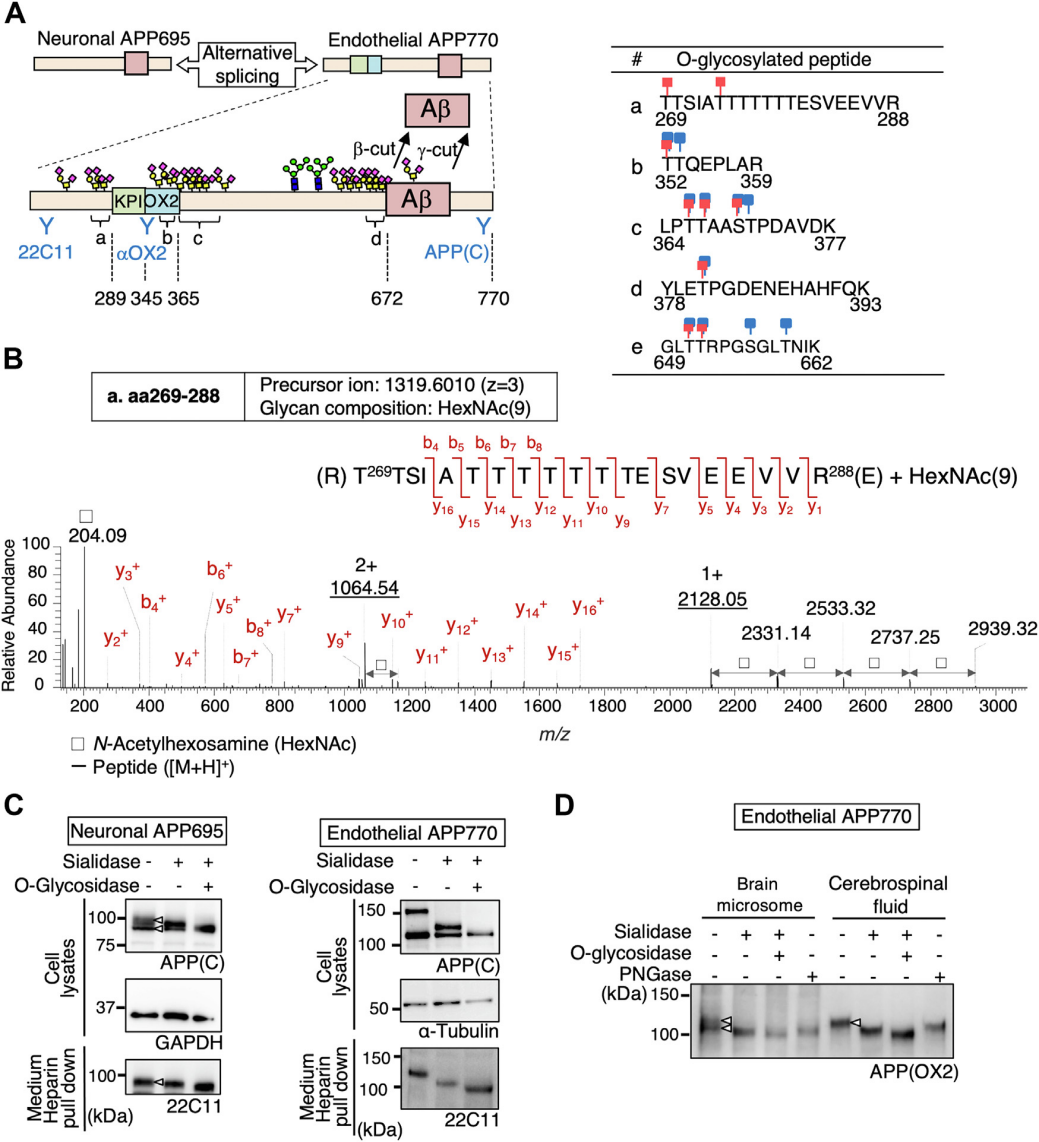
**O-glycosylated APP is preferentially secreted.***A*, schematic of neuronal APP695 and endothelial APP770 structure, in the latter of which KPI and OX2 domains, N- and O-glycosylation sites and site-specific anti-APP antibodies are shown. The positions of identified O-glycosylation peptides are also shown (*A*–*D*). The table shows peptide sequences with known O-glycosylation sites (*blue*) and the O-glycosylation sites *identified in this study* (*red*)(Fig. S1). *B*, product ion spectrum of APP770 O-glycopeptide (aa269–288) arising from the precursor ion at *m/z* 1319.6010 (z = 3). The oxonium ion at *m/z* 204.09 represents N-acetyl hexosamine (HexNAc). b-ion: Fragment ion containing the peptide N-terminus formed upon dissociation of a peptide ion at the peptide backbone C–N bond. y-ion: Fragment ion containing the peptide C-terminus formed upon dissociation of a peptide ion at the peptide backbone C–N bond. *C*, lysates and sAPP pulled down with heparin agarose from the medium of mouse primary neurons and BMECs were treated with sialidase or O-glycosidase and analyzed by immunoblotting for APP and GAPDH and anti-α-tubulin (loading control). *D*, human brain microsomes and cerebrospinal fluid were incubated with heparin agarose to pull down APP and sAPP, respectively. The samples were treated with sialidase, O-glycosidase, or PNGase, and blotted for APP770 and sAPP770, respectively.

Only limited information is available concerning APP O-glycosylation sites, and therefore we first conducted site-specific mapping of APP O-glycans using a mass spectrometry-based method. Hemagglutinin (HA)-tagged human APP770 was expressed in HEK293T cells, and HA-sAPP770 purified from culture medium was treated with trypsin plus OpeRATOR protease, the latter of which specifically cleaves N-terminally O-GalNAc glycan-occupied Ser and Thr residues (18) and used for MS/MS analysis. In addition to several known O-glycosylation sites (9, 10), we additionally identified Thr269 and Thr274 as novel O-glycosylation sites (Figs. 1, *A* and *B* and S1). Notably, O-glycosylation sites of APP are concentrated at two sites: one near the KPI plus OX2 domain and the other near the β-cleavage sites.

We then analyzed mouse neuronal APP695 and endothelial APP770, and Western blot analysis using anti-C-terminal APP antibody revealed that full-length APP exhibits double bands in both types of cell lysate (Neuronal APP695 gives ∼100 kDa signal and endothelial APP770 gives 120∼150 kDa signal, respectively). After treatment with sialidase plus O-glycosidase, the latter being an enzyme that specifically removes non-sialylated core one O-glycan disaccharide (Galβ1-3GalNAcα1-Ser/Thr), in both cell types the upper APP band disappeared and merged with the lower band (Fig. 1*C*), indicating that the upper band represented sialylated core 1-type O-glycosylated APP and the lower band represented hypo-O-glycosylated APP. Because sAPP released into the media showed poor reactivity to most anti-APP antibodies, heparin-agarose was alternatively used to pull down sAPP by virtue of APP having two heparin-binding domains in its extracellular domain. sAPP released into the medium from both types of cells was most sensitive to sialidase plus O-glycosidase, indicating that sAPP was mostly O-glycosylated. We then extended this analysis to human brain samples. Brains possibly contain a mixture of different APP isoforms (APP695, 751, and 770), making analysis difficult. We therefore used anti-OX2 antibody that specifically recognizes APP770 (Fig. 1*A*) and focused on endothelial APP770, which is heavily O-glycosylated and thus easily differentiated from its hypo-O-glycosylated form by SDS-PAGE. Again, the human brain microsome fractions contained both forms of APP770, whereas the sAPP770 in the cerebrospinal fluid (CSF) lacked the hypo-O-glycosylated form (Fig. 1*D*).

### Endothelial cell surface APP770 receives O-glycan sialylation

**Figure 2:**
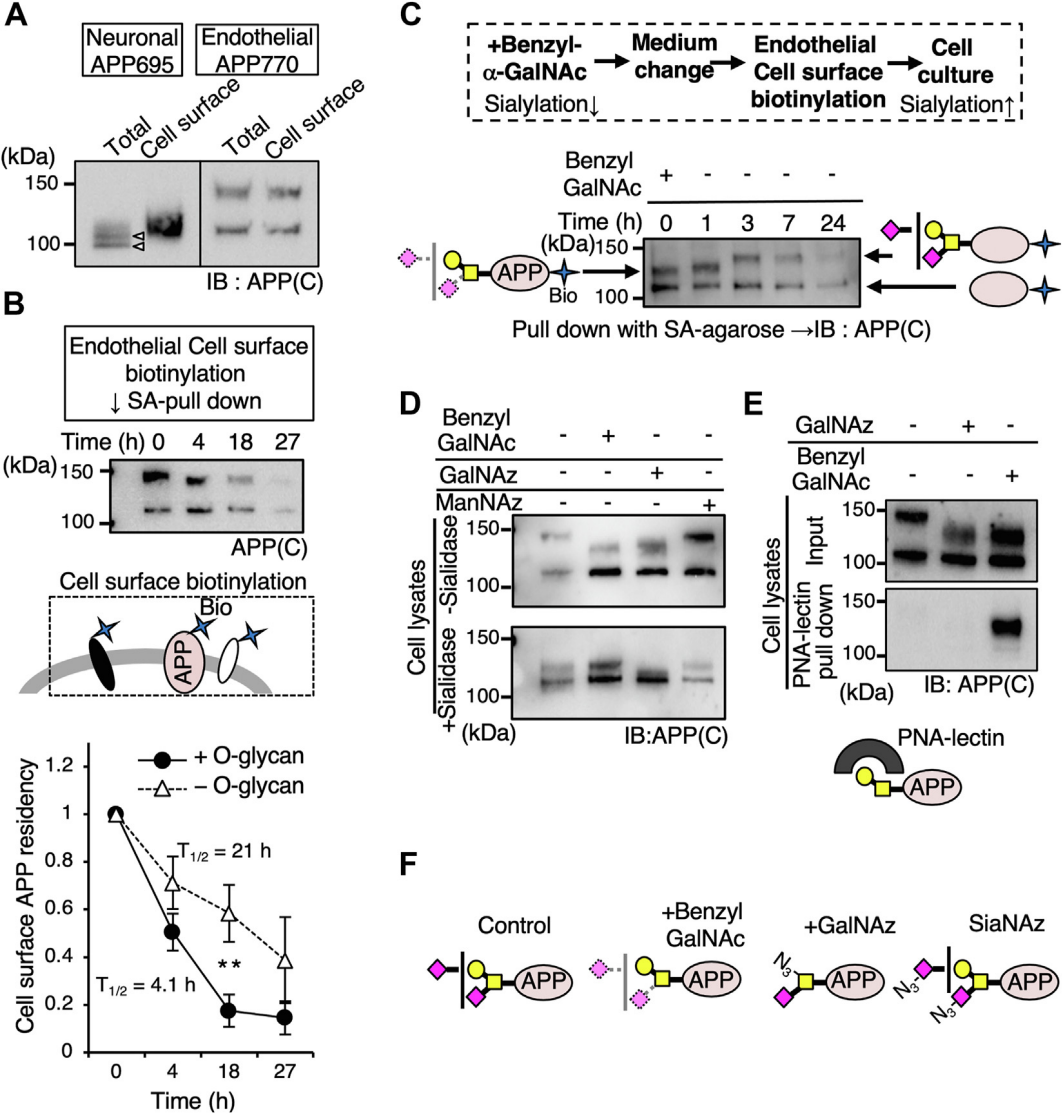
**Cell surface APP is internalized and its O-glycans are sialylated.***A*, after cell surface labeling with NHS-LC-biotin and subsequent incubation of mouse primary neurons and human brain microvascular endothelial cells (BMECs), biotinylated proteins were pulled down with streptavidin (SA)-agarose and blotted for APP. *B*, after cell surface labeling with sulfo-NHS-LC-biotin and subsequent incubation, biotinylated proteins were pulled down with SA-agarose and blotted for APP. On the basis of the quantification of biotinylated APP, the half-life of cell surface APP was expressed as mean ± SEM (n = 3 independent western blots). ∗∗*p* < 0.01, Student’s *t* test. *C*, after BMECs were incubated with benzyl-α-GalNAc for 16 h, the cells were surface-biotinylated and cultured again in the absence of benzyl-α-GalNAc for different periods. Biotinylated proteins pulled down with SA-agarose were blotted for APP. Possible O-glycan structure with APP in the *upper* band before and after benzyl-α-GalNAc treatment is shown. *D*, BMECs were cultured with benzyl GalNAc, Ac_4_GalNAz, or Ac_4_ManNAz, and the cell lysates were treated with sialidase and blotted for APP. The upper band of all samples was sensitive to sialidase, indicating that GalNAz incorporation allowed sialylation. *E*, BMECs were cultured with benzyl-α-GalNAc or Ac_4_GalNAz. The cell lysates were incubated with immobilized PNA lectin that detects the Galβ1,3GalNAc structure. PNA lectin-precipitated samples were then blotted for APP. GalNAz-incorporated APP was not reactive with PNA lectin, indicating impaired galactosylation. *F*, considering the results in (*D*) and (*E*), typical O-glycan structure of APP, treated with benzyl-α-GalNAc, GalNAz, or ManNAz (for SiaNAz), is shown.

It was considered that the unique feature of APP of O-glycosylated APP and hypo-O-glycosylated APP (12) being separated by SDS-PAGE would provide us with a unique opportunity to clarify the relationship between the O-glycosylation pathway and intracellular APP trafficking. We first performed a cell surface biotinylation experiment using neurons and endothelial cells. In neurons, only the upper band was biotinylated, indicating that APP695 was transported to the cell surface after O-glycosylation (Fig. 2*A*). Unexpectedly, however, in endothelial cells not only O-glycosylated but also hypo-O-glycosylated APP770 was biotinylated. These results indicate that, in neurons, fully glycosylated APP695 is selectively transported to the cell surface, while in endothelial cells, APP770 can be transported to the cell surface with fewer O-glycans. The latter result contradicts the accepted belief that nascent proteins receive O-glycans in the Golgi apparatus before reaching the cell surface (19) and does not fully explain why sAPP is exclusively O-glycosylated. One possibility, namely, that hypo-O-glycosylated APP770 could be less stable than O-glycosylated APP770 and only the latter survives was ruled out by half-life analysis, as the half-life of cell surface biotinylated hypo-O-glycosylated APP770 was ∼21 h, which was ∼5 times longer than that of O-glycosylated APP770 (4.1 h) (Fig. 2*B*).

Another possibility is that cell surface hypo-O-glycosylated APP770 is internalized and undergoes O-glycosylation within the Golgi apparatus before processing. To explore this possibility, we designed an experiment that used a combination of cell surface biotinylation and treatment of cells with benzyl-α-GalNAc, which act as an effective surrogate substrate and inhibits the extension of O-glycans at high concentrations (2–4 mM), such as the sialylation of core 1-type O-glycan (20, 21). After treatment, the benzyl-α-GalNAc was removed from the medium and the cells were labeled with biotin. Cell surface biotinylated proteins were enriched by streptavidin (SA)-agarose. We first checked that the molecular weight of biotinylated O-glycosylated APP770 was reduced by this treatment (Fig. 2*C*) (12). Indeed, the benzyl-α-GalNAc-treated APP770 was hyposialylated (Fig. 2*D*), but had Galβ1,3GalNAc residues, based on the reactivity to PNA lectin (Fig. 2, *E* and *F*). Interestingly, further incubation in the absence of benzyl-α-GalNAc led to an increase in the molecular weight of biotinylated O-glycosylated APP770 (Fig. 2*C*). These results indicate that cell surface APP770 is internalized and then modified with sialic acid to produce extended O-glycans.

### Hypo-O-glycosylated APP770 at the endothelial cell surface is internalized and modified by O-glycans

**Figure 3:**
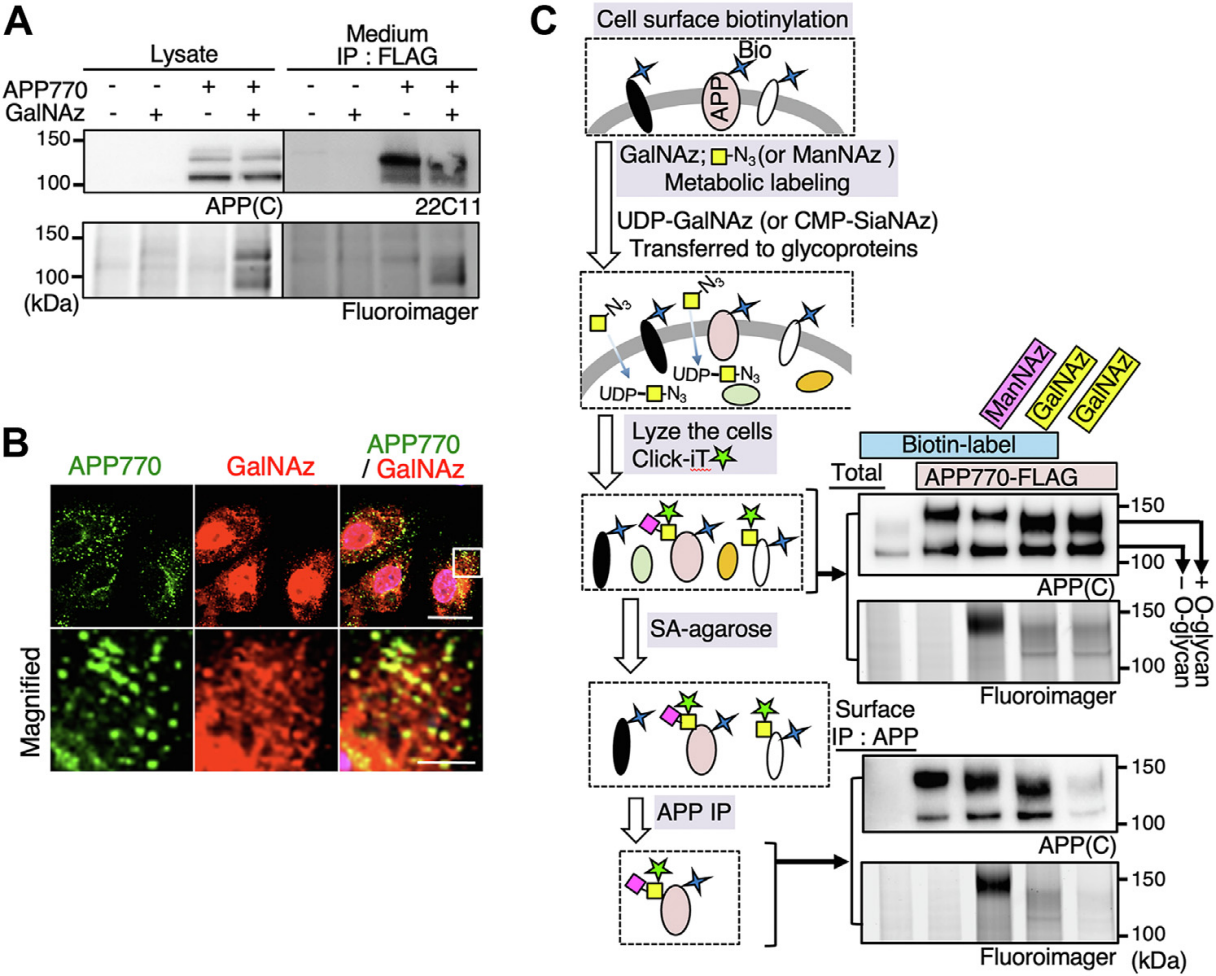
**Endothelial cell surface APP770 is internalized and O-glycosylated.***A*, BMECs expressing FLAG-APP770 with an adenovirus system were metabolically labeled with GalNAz. The cell lysates were treated with TAMRA-conjugated DIBO, and sAPP pulled down with anti-FLAG (M2)-coupled agarose was blotted for APP770 or analyzed by a fluoroscanner. *B*, immunofluorescence microscopy shows that endothelial APP770, detected with anti-OX2 antibody, co-localizes with O-GalNAz glycan signals. Scale bar, 20 μm. *Lower panels* show magnified images. Scale bar, 5 μm. *C*, after cell surface biotinylation, cells were cultured with Ac_4_GalNAz or Ac_4_ManNAz for 6 h, and the lysates were then treated with TAMRA-conjugated DIBO to fluorescently label the azide group. The presence of O-GalNAz glycan in the biotinylated APP was assessed by a fluoroscanner.

We then hypothesized that the initial O-GalNAc transfer event might also occur upon the internalization of endothelial cell surface APP770. GalNAc and sialic acid residues can be metabolically labeled using peracetylated azide sugars, Ac_4_GalNAz and Ac_4_ManNAz, which are incorporated into O-glycans as GalNAz and SiaNAz, respectively, *via* endogenous biosynthetic pathways, and can be covalently tagged with an azide-reactive probe (22, 23). Thus, we performed cell surface biotinylation and subsequent O-glycan metabolic labeling. Using an adenovirus technique, APP770-FLAG was expressed in endothelial cells, which were treated with Ac_4_GalNAz or Ac_4_ManNAz (24, 25). The incorporated azide sugars were click-labeled with TAMRA-conjugated dibenzocyclooctyne (DIBO) (26). We detected fluorescent signals corresponding to the O-GalNAz glycosylated APP770-FLAG, verifying the incorporation of GalNAz into APP770 (Fig. 3*A*). The incorporation of GalNAz into APP770 was also examined by mass spectrometry analysis of the immunopurified APP770-derived glycopeptide (Fig. S2). Furthermore, analysis with immunofluorescence microscopy revealed that most of the intracellular APP770 signals co-localized with GalNAz signals (Fig. 3*B*). Notably, part of GalNAz is enzymatically converted to UDP-GlcNAz in addition to UDP-GalNAz and nuclear O-GlcNAcylated proteins are presumably O-GlcNAz-labeled (27). Indeed, in the mutant CHO cell line, IdlD cells, which lack UDP-galactose epimerase (GALE) activity and are unable to convert GalNAz to GlcNAz, we found no nuclear azide signal, with all of the signals instead being found in the intracellular vesicles (Fig. S3) (27). Next, O-GalNAz glycans as well as APP770 were visualized with several organelle markers in endothelial cells. As has been reported in other cells (28, 29), APP co-localized with the trans-Golgi marker adaptin-γ, the recycling endosome marker Rab11, the early endosome markers EEA1 and Rab5, and, to a lesser extent, the late endosome marker Rab7 (Fig. S4, *A* and *B*). In comparison with APP770, the O-GalNAz glycan signal co-localized less with adaptin-γ and EEA1 but was relatively enriched in the recycling endosome (Fig. S4, *C* and *D*). Next, we combined the cell surface biotinylation experiment with metabolic sugar labeling. After cell surface biotinylation and subsequent metabolic labeling with Ac_4_GalNAz or Ac_4_ManNAz, we found that each biotinylated APP770 had a fluorescent signal derived from azide sugars (Fig. 3*C*), clearly demonstrating that cell surface biotinylated APP770 was internalized and then underwent O-GalNAz and SiaNAz glycan modifications. Notably, the molecular weight of GalNAz-labeled APP770 was lower than that of wild-type APP770. Based on the analysis of GalNAz-labeled APP770 with sialidase and PNA lectin, we found that GalNAz incorporation allowed sialylation but blocked subsequent galactosylation (Fig. 2, *D*, *E* and *F*). However, sAPP770 was still generated and fluorescently detected in the culture medium (Fig. 3*A*), indicating that the Ac_4_GalNAz treatment did not impair the overall intracellular trafficking of APP770.

### O-glycosylation of APP770 affects Aβ generation

**Figure 4:**
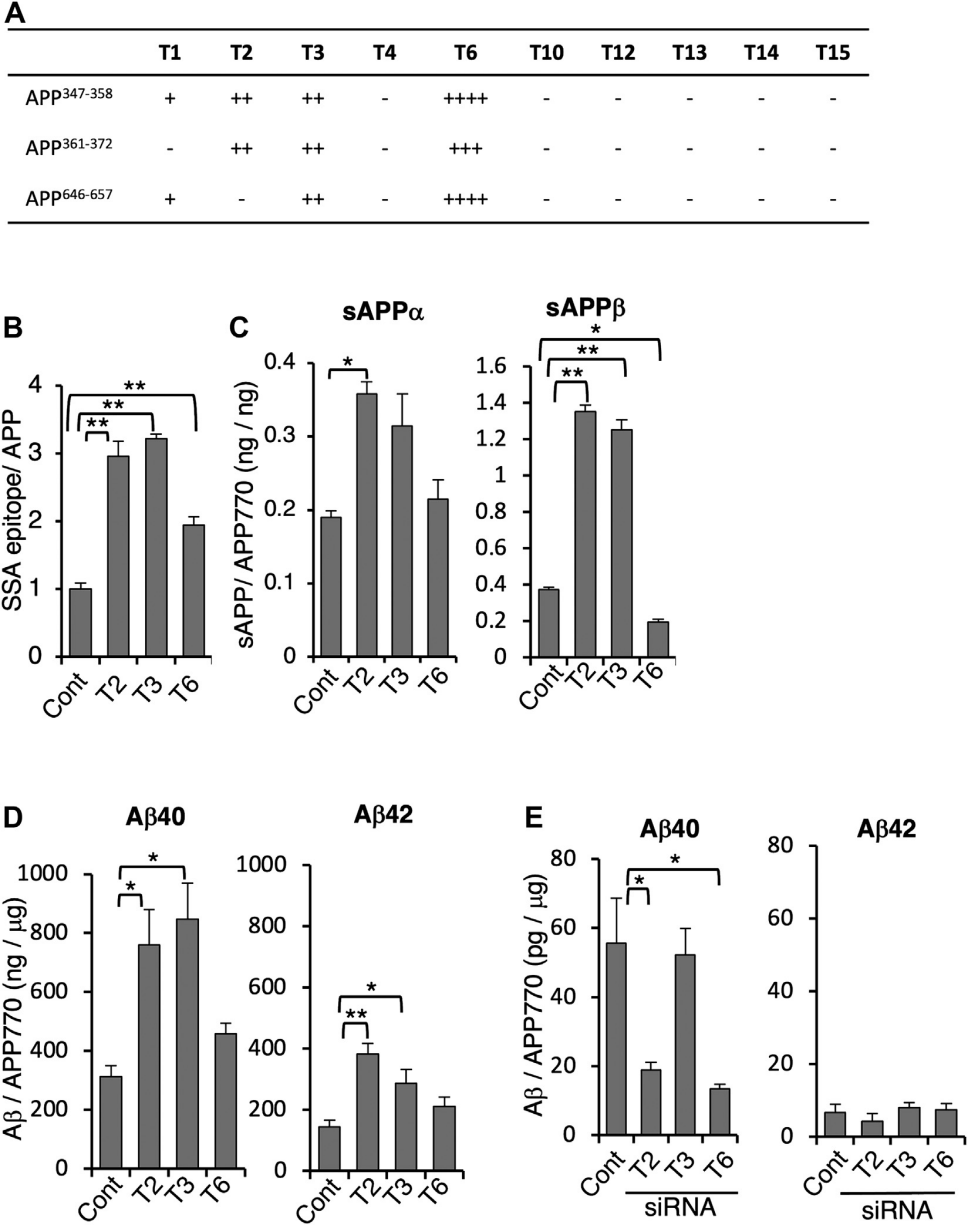
**Modulation of the APP O-glycosylation enzyme affects Aβ generation.***A*, three kinds of APP-derived peptides were incubated with a series of GalNAc-T enzymes and UDP-GalNAc, and the reaction products were analyzed by MS. Incorporation of GalNAc was roughly quantified as the ratio of the signal intensity of the GalNAc-incorporated peptide to the sum of signal intensities for the acceptor peptide plus GalNAc-incorporated peptide as follows; not detectable: −, less than 5%: +, 5 to 25%: ++, 25 to 50%: +++, 50 to 75%: ++++. *B*, lysates of BMECs transfected with GalNAc-T2, -T3, -T6, or control vector were analyzed by SSA lectin ELISA and APP770 levels to measure O-glycans on APP. Data show mean ± SEM, n = 3. *C* and *D*, BMECs were transfected with GalNAc-T2, -T3, -T6, or control vector. The levels of sAPPα and sAPPβ in the medium (*C*) or intracellular Aβ40/42 (*D*) were measured and are shown as the mean ± SEM, n = 6. *E*, HeLa cells were transfected with GalNAc-T2, T3, and T6 or control siRNA. The levels of intracellular Aβ40 and 42 were measured and are shown as mean ± SEM. Statistical analysis was mainly performed by one-way ANOVA with Dunnett’s multiple comparison test or Tukey-Kramer test (for d, Aβ42); ∗*p* < 0.05, ∗∗*p* < 0.01.

Although endothelial APP770 is transported to the cell surface in an O-glycosylation-independent manner, sAPP is exclusively O-glycosylated, raising the possibility that the level of O-glycan in APP770 could affect its processing. Twenty polypeptide GalNAc-T (GalNAc-T) genes have been identified as being involved in the initiation of O-glycosylation of proteins in humans (19). First, to identify the responsible GalNAc-T enzyme(s), three kinds of APP770-derived peptides, which are reported to be O-glycosylated by several groups (9, 10) including ours, were incubated with a series of recombinant GalNAc-Ts and UDP-GalNAc *in vitro*, and the reaction products were analyzed by mass spectrometry (30). In addition to GalNAc-T2 and -T3, which have been previously reported to transfer GalNAc to APP770 (9), we found that GalNAc-T6, which has the highest homology with GalNAc-T3 (Fig. S5*A*) (19), could also transfer GalNAc to all of the peptides (Figs. 4*A* and S5*B*) (19). Other GalNAc-Ts exhibited negligible activity. Notably, we could even detect the product in which two GalNAc residues are incorporated into the APP^361–372^ peptide by GalNAc-T6. However, GalNAc-T4, -T12, and -T14, which have higher homology with GalNAc-T2, -T3, and -T6, showed no activity with any of the peptide substrates, suggesting that GalNAc-T2, -T3, and -T6 are the major APP O-glycosylation enzymes.

To define whether or not these GalNAc-Ts act on APP770 O-glycosylation in the cell, we developed an APP770 sandwich lectin ELISA system with *Sambucus sieboldiana* agglutinin that detects α2,6-sialylated O-GalNAc glycan. The overexpression of GalNAc-T2, -T3, and -T6 markedly increased the level of sialylated O-glycan on APP770 (Fig. 4*B*). We then investigated the effect of APP O-glycosylation on APP processing. In addition to sAPPα/β, we focused on the production of Aβ40 and Aβ42, both of which are typical Aβ species, and a lower concentration of Aβ42 and a lower ratio of Aβ42 to Aβ40 in CSF are associated with AD brain pathology (31). Overexpression of GalNAc-T2 and -T3 resulted in significant increases in sAPPα (∼2-fold), sAPPβ (∼3-fold) (Fig. 4*C*), and Aβ40/42 (∼2-fold) (Fig. 4*D*). GalNAc-T6 exhibited higher enzyme activity to APP770 *in vitro*, whereas overexpression of GalNAc-T6 did not increase the secretion of sAPP. Previous reports showed that GalNAc-T6 overexpression reduces the level of cell adhesion molecules, such as E-cadherin and fibronectin (32), and a reduction in these cell adhesion molecules could affect intracellular APP sorting. Partial knockdown of GalNAc-T2 and -T6, but not -T3, in HeLa cells significantly reduced the cellular level of Aβ40 but not Aβ42 (Fig. 4*E*). Taken together, these findings indicate that APP O-glycosylation regulates APP processing.

### Internalized APP770 encounters O-GalNAc enzymes in the Golgi apparatus

**Figure 5:**
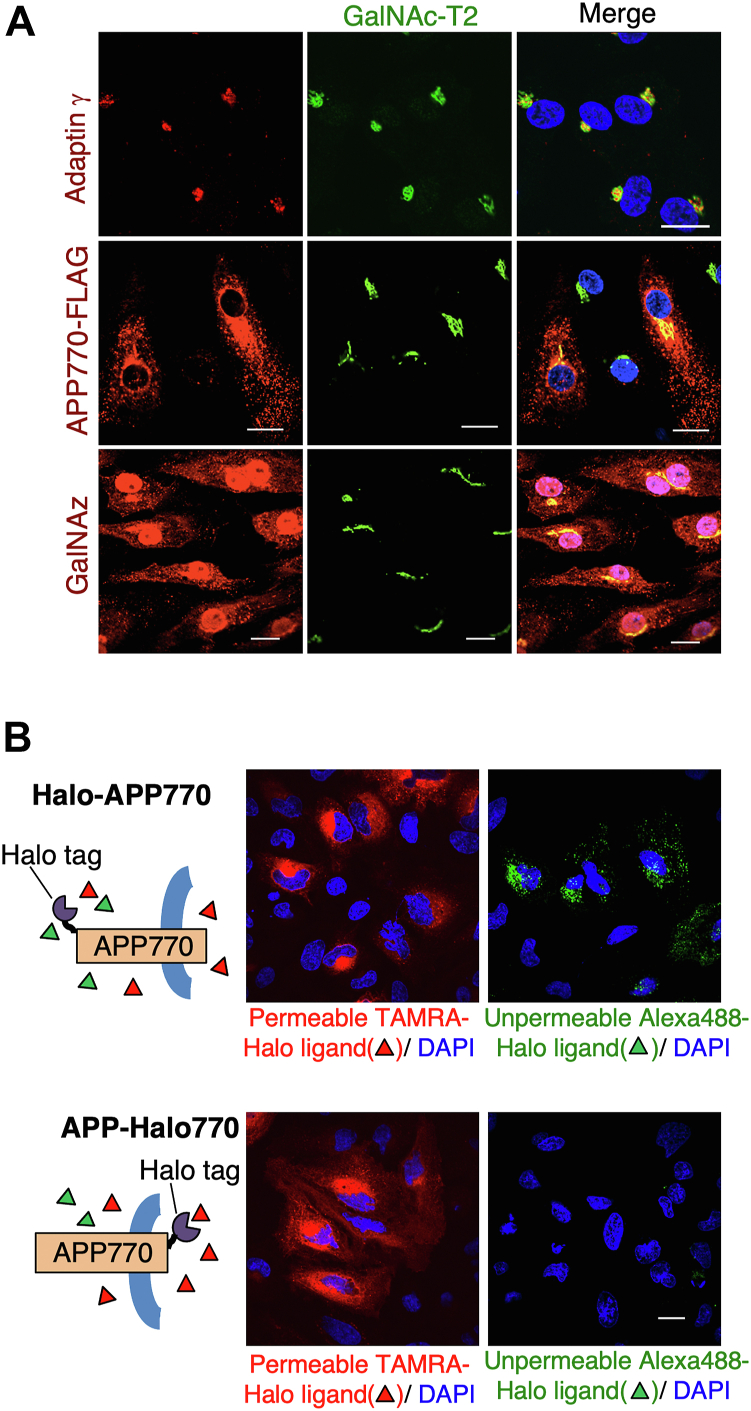
**O-glycosylation enzymes co-localize with GalNAz and internalized APP770.***A*, BMECs expressing APP770-FLAG were cultured with GalNAz for 6 h. Following fixation, the cells were reacted with Alexa555-labeled alkyne. Immunostaining analysis of GalNAc-T2 was performed for adaptin-γ, APP770-FLAG, and GalNAz. Scale bar, 20 μm. *B*, HeLa cells expressing Halo-APP770 or APP70-Halo were reacted with the non-permeable HaloTag Alexa488 ligand or the permeable HaloTag TAMRA ligand. Scale bar, 20 μm.

Immunofluorescence microscopic analysis confirmed that GalNAc-T2 mostly co-localized with the trans-Golgi marker adaptin-γ (11, 19) and overlapped with APP770 and O-GalNAz signals (Fig. 5*A*). We, therefore, expected that the internalized APP770 would be transported to the Golgi apparatus for its O-glycan modification. To investigate this, we fused APP770 with Halo Tag protein, a 297 residue self-labeling protein tag. Just before fixing the cells, a synthetic HaloTag ligand (Fluorescent or biotin type) was added to the media for a short period (5–30 min) to specifically label the cell surface Halo-tagged APP. Covalent binding between Halo-APP770 and its ligand would enable us to observe the internalized APP770. We first prepared two types of Halo-tagged APP770, Halo-APP770, in which the N-terminal extracellular domain was tagged with Halo, and APP770-Halo, in which the C-terminal cytoplasmic region was tagged with Halo, and determined that both could be labeled with membrane-permeable HaloTag TAMRA ligand (Fig. 5*B*). In contrast, only Halo-APP770 was labeled with the non-permeable HaloTag Alexa488 ligand, indicating the feasibility of analyzing the fate of cell surface APP770 after internalization. Furthermore, Alexa488 ligand-conjugated soluble Halo-APP770 was detected in the media, indicating that Halo-APP770 was properly transported in the cells just like wild type APP.

**Figure 6:**
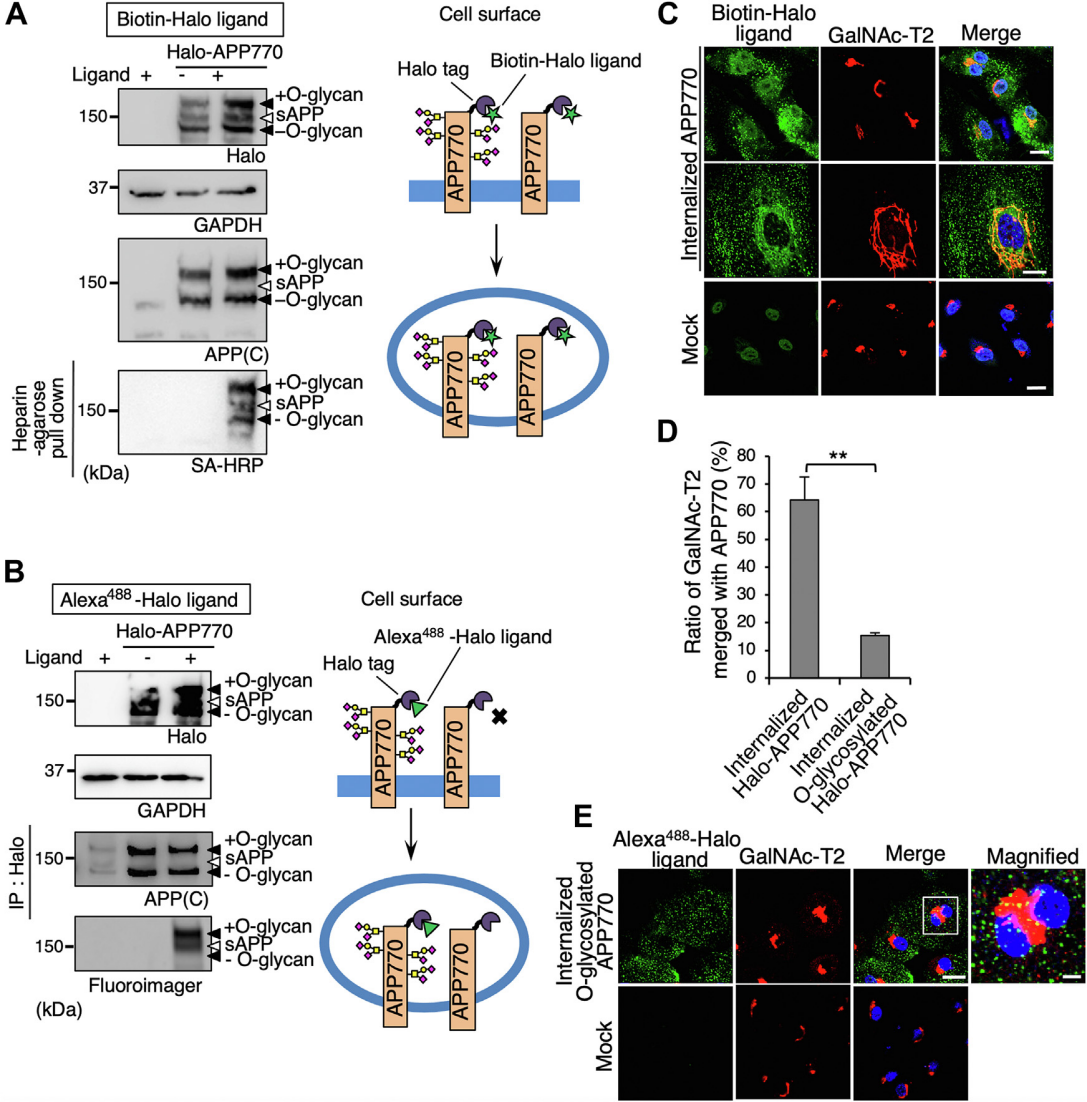
**Internalized non-O-glycosylated APP770 is transported to the Golgi apparatus to encounter GalNAc-T2.***A*–*E*, BMECs expressing Halo-APP770 with an adenovirus system were treated with HaloTag biotin ligand and Alexa488-labeled SA (*A* and *C*) or HaloTag Alexa488 ligand (*B* and *E*). *A* and *B*, the cell lysates and sAPP pulled down with heparin-Sepharose from the culture medium were analyzed by Western blot for APP or HaloTag ligand-coupled APP770. *C* and *E*, for immunofluorescence, the cells were fixed and immunostained for GalNAc-T2. Scale bars, 20 μm and 5 μm (for magnified images). *D*, on the basis of the images shown in (*C*) and (*E*), the area of co-localization of GalNAc-T2 with internalized Halo-APP770 was determined as mean ± SEM. ∗∗*p* < 0.01, Student’s *t* test.

During biochemical analysis of internalized Halo-APP770, we unexpectedly found that non-permeable HaloTag Alexa488 and biotin ligands exhibited different binding activity to internalized Halo-APP770. The HaloTag biotin ligand bound to both O- glycosylated and hypo-O-glycosylated APP770 (Fig. 6*A*), whereas the HaloTag Alexa488 ligand bound almost exclusively to the O-glycosylated APP770 (Fig. 6*B*). These results indicated that, using these ligands, internalized O-glycosylated APP770 (Alexa488-Halo ligand) could be discriminated from internalized APP770 overall (Biotin-Halo ligand). Notably, the internalized APP significantly co-localized with GalNAc-T2 (Fig. 6, *C* and *D*) (33), while internalized O-glycosylated APP770 exhibited poor co-localization with GalNAc-T2 enzyme (Fig. 6, *D* and *E*). These results suggest that internalized hypo-O-glycosylated APP770 is transported to the Golgi for O-glycosylation, after which it leaves the Golgi and is transported to different locations.

## Discussion

**Figure 7:**
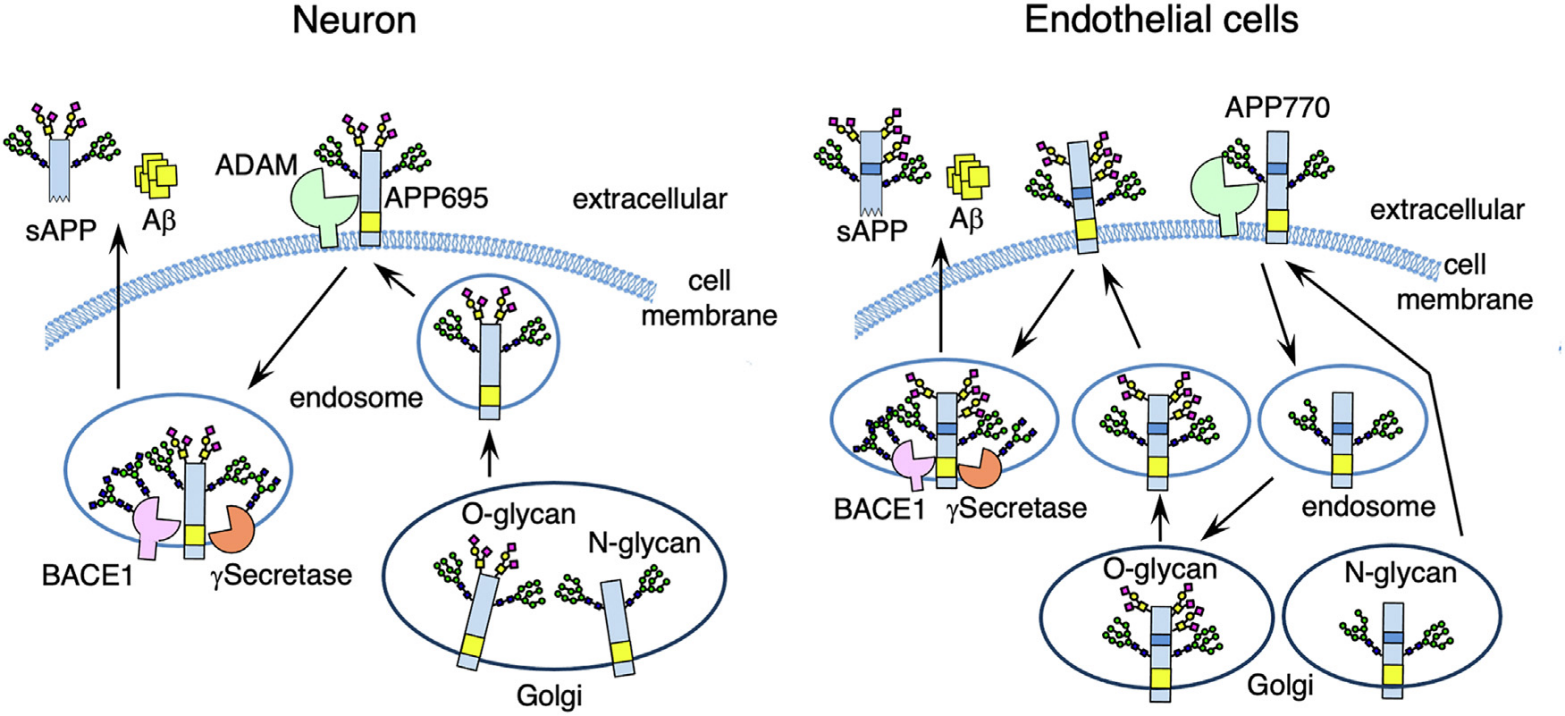
**Intracellular trafficking and glycosylation of APP differ among cell types.** In neurons, both N- and O-glycosylated APP695 are transferred to the cell surface, then internalized APP undergoes processing by BACE one and γ-secretase to generate Aβ. In the endothelial cells, N-glycosylated APP770 is transported to the cell surface regardless of O-glycosylation. Non-O-glycosylated APP770 is internalized and transported in a retrograde fashion to the Golgi apparatus where it undergoes O-glycosylation. The O-glycosylated APP770 exhibits decreased co-localization with Golgi-resident O-glycosylation enzymes and is eventually processed to generate Aβ.

In protein glycosylation, it is generally believed that newly synthesized proteins undergo both N- and O-glycosylation before being delivered to the cell surface. However, several recent reports show exceptions to this classical glycosylation pathway. The epithelial mucin, MUC1, is constitutively internalized and sialylated during recycling (34). Another example is an extrinsic sialylation in which IgG and other serum glycoproteins are sialylated by serum-localized nucleotide sugar donor CMP-sialic acid, which has been reported by several groups (35, 36). In this study, we demonstrated another non-classical glycosylation pathway that regulates intracellular APP trafficking in endothelial cells. We found that, in neurons, both N- and O-glycosylated APP695 are transferred to the cell surface, while in endothelial cells, hypo-O-glycosylated APP770 arrives at the cell surface but is then internalized for retrograde transport to the Golgi apparatus where it undergoes O-glycosylation (Fig. 7). By using two kinds of HaloTag ligand, we coincidentally succeeded in differentiating the internalization of O-glycosylated APP770 from that of hypo-O-glycosylated APP770. However, the reason why the bulky and hydrophobic HaloTag Alexa488 ligand binds exclusively to the O-glycosylated Halo-APP770 remains unclear. We found that the internalized APP770 significantly co-localized with O-glycosylation enzymes in the Golgi, while internalized O-glycosylated APPs exhibited markedly less co-localization to these O-glycosylation enzymes. Moreover, immunofluorescent microscopy showed that, compared with the intracellular APP770 signal, the O-GalNAz glycan signal revealed less Golgi localization. These findings suggest that internalized hypo-O-glycosylated APP770 is transported in a retrograde fashion to meet the Golgi O-glycosylation enzymes, and fully O-glycosylated APP770 leaves the Golgi and is transferred differently for processing. An impaired endocytic pathway is implicated in AD, and several molecules, such as low-density-lipoprotein receptor (LDLR) family proteins and PICALM, are reported to be possible sorting receptors for APP and Aβ (5, 37). One interesting possibility is that these sorting receptors recognize the O-glycosylation status of APP *via* as-yet-undefined glycan recognition molecules.

Although our *in vitro* and cell-based analyses show that GalNAc-T2 is the enzyme responsible for endothelial APP O-glycosylation, GalNAc-T2 siRNA led to a partial reduction in Aβ. GalNAc-T3 and T6 activities could O-glycosylate APP alternatively, almost enough for Aβ production. Furthermore, apoC-III and LRP1, have been reported as physiological substrates of GalNAc-T2 (11), and it is unclear whether GalNAc-T2 inhibition could impair the functionality of these substrates, leading to disturbed lipid metabolism and vesicle sorting. Moreover, the overexpression of GalNAc-T6 did not increase the secretion of sAPP. A previous study has reported that GalNAc-T6 overexpression reduces the level of E-cadherin (32), and a reduction in such a cell adhesion molecule could also affect the intracellular sorting machinery.

As another important aspect of APP O-glycosylation, we could only observe an effect of GalNAc-T overexpression on the production of sAPP770 and Aβ in cells cultured in low-glucose medium (∼5 mM), in which the level of UDP-GalNAc was limited (38). Given that UDP-GlcNAc is the end product of the hexosamine pathway and GALE effectively converts UDP-GlcNAc to UDP-GalNAc (39), the concentrations of UDP-GalNAc and UDP-GlcNAc are highly sensitive to ambient glucose levels (40). Therefore, it is possible that the GalNAc-T enzymes as well as their donor substrate levels could critically regulate Aβ generation.

## Experimental procedures

### Materials

The sources of the materials used in this study were as follows: pCALNL5 (RDB01862, RIKEN BioResource Center), a series of GalNAcT (1, 2, 3, 4, 6, 10, 12, 13, 14, and 15)-pcDNA3.1neoGW plasmids (National Institute of Advanced Industrial Science and Technology), tissue culture medium and reagents, including Dulbecco’s modified Eagle’s medium (DMEM) from Invitrogen; protein G-Sepharose Fast Flow and immobilized streptavidin Mutein Matrix from Roche; protein molecular weight standards from Bio-Rad; BCA protein assay reagents and sulfo-NHS-LC-biotin and sulfo-NHS-SS-biotin from Thermo Fisher Scientific Inc; and all other chemicals from Sigma or Wako Chemicals. HaloTag Alexa Fluor 488, HaloTagPEG biotin and HaloTag TAMRA ligands, pFC14K HaloTag CMV Flexi vector, and anti-HaloTag monoclonal antibody were from Promega. Other commercially available antibodies used were anti-APP (mouse, 22C11, Chemicon), anti-sAPPα (mouse, 6E10, BioLegend), anti-human APP770 (rabbit, Japan-IBL), anti-EEA1 and anti-adaptin-γ (mouse, BD Transduction Laboratories), Alexa Fluor 488-anti-Aβ (6E10, BioLegend), anti-PECAM (rat, MEC13.3, BioLegend), anti-tubulin β3 (mouse, TUJ1, BioLegend), anti-CD146 (rat, ME-9F1, BioLegend) anti-O-GlcNAc (RL2) (mouse, Thermo Fisher Scientific Inc), anti-GAPDH (mouse, MAB374, Chemicon), anti-α-tubulin (mouse, Sigma), anti-FLAG (M2) (mouse, Sigma), anti-ppGalNAc-T2 (rabbit, Sigma), anti-ppGalNAc-T3 (sheep, R&D Systems), anti-ppGalNAc-T6 (rabbit, Abcam), anti-Rab5 and anti-Rab7 (rabbit, Cell Signaling Technology), anti-APP (APP(C)), anti-APP OX2, and anti-human sAPPβ (rabbit, IBL-Japan).

### Construction of plasmids

APP770FLAG-pcDNA was generated as described previously (12). For the APP770 Halo expression vector, the APP portion (forward, 5′-*gcgtacgc*ATGCTGCCCGGTTTGGCACTGCTC-3′, and reverse, 5′-*gatatc*CGTTCTGCATCTGCTCAAAGAAC-3′) was inserted into the pFC14K HaloTag CMV Flexi vector. A series of adenovirus vectors was produced using the ViralPower Adenoviral Gateway Expression kit (Life). pENTER-FLAG APP770 was constructed by inserting the N-terminal signal peptide of APP770 (forward, 5′-*gtagac*ATGCTGCCCGGTTTGGCACTGC-3′, and reverse, 5′-*aagctt*CGCCCGACCGTCCAGGCGG-3′) and the remaining part of APP770 (forward, 5′-*tctaga*CTGGAGGTACCCACTGATG-3′, and reverse, 5′- *gcggccgc*CTAGTTCTGCATCTGCTCAAAG-3′) into the FLAG-containing pBluescript (12), then transferred into the pENTER plasmid. The pENTER-Halo APP770 construct was created by replacing the FLAG sequence of FLAG-APP770 with the Halo portion (forward, 5′-*aagctt*GGATCCGAAATCGGTACTGGCTTTC-3′, and reverse, 5′-*tctaga*ACCGGAAATCTCCAGAGTAGACAGCC-3′). For pENTER GalNAcT constructs, the following primers were used: GalNAcT2 forward, 5′-CACCATGCGGCGGCGCTCGCG-3′ and reverse, 5′-CTGCTGCAGGTTGAGCGTGAA-3′; GalNAcT3 forward, 5′-CACCATGGCTCACCTAAAGCGACTAGTAAAA-3′ and reverse, 5′-*ggattc*ATCATTTTGGCTAAGTATCCATTTTTG-3′; and GalNAcT6 forward, 5′- CACCCTCGAGATGAGGCTCCTCCGCAGACG-3′ and reverse, 5′-GACAAAGAGCCACAACTGATGG-3′. pAd-GalNAcT2, 3, and six were then generated.

### Mice

All animal experiments were performed in compliance with RIKEN’s Institutional Guidelines for Animal Experiments.

### Cell culture, expression plasmids, and RNA interference

Human brain microvascular endothelial cells (BMECs, Applied Cell Biology Research Institute) and human umbilical vein endothelial cells (HUVECs, TaKaRa Bio Inc) were, respectively, cultured in CS-C complete medium (Cell Systems) and Endothelial Cell Basal Medium 2 (TaKaRa Bio Inc) with FBS and used within four passages. Mouse primary liver sinusoidal endothelial cells (41) and neurons (8) were isolated and cultured as previously reported. HeLa, SK-NSH, CHO (RIKEN Cell Bank), or its mutant IdlD cells were maintained in high-glucose DMEM containing 10% FBS. For biochemical experiments, both BMECs and HeLa cells were cultured in low-glucose conditions, with MCDB131 (Sigma-Aldrich) and DMEM containing 10% FBS, respectively, for at least 24 h. The BMECs were then transfected using Nucleofector (Lonza, Basi Nucleofector Kit for primary endothelial cells, program M003), and the HeLa cells were transfected using FuGENE6 reagent (Promega). For knockdown experiments, Stealth RNAis (Invitrogen) were used. HeLa cells at 50% confluency on 6-cm dishes were infected with hAPP770FLAG-pAd. After 24 h, the cells were transfected with 50 pmol control siRNA (Stealth RNAi negative control medium GC Duplex) or siRNA for GalNAcT2 (HSS103983), GalNAcT3 (HSS103984), or GalNAcT6 (HSS117436) using Lipofectamine RNAiMAX Transfection Reagent (Thermo Fisher Scientific). After 16 h, the culture medium was changed to a low-glucose DMEM medium containing 2% FBS. After 24 h, the cells and medium were collected for further analysis.

### Human samples

The clinical study was approved by the ethical committees of RIKEN, Tokyo Metropolitan Institute of Gerontology, Tokyo Metropolitan Geriatric Hospital, and Fukushima Medical University. Frozen tissues from the postmortem brain were obtained from the Brain Bank for Aging Research, which consists of samples from consecutive autopsy cases from a general geriatric hospital with informed consent obtained from the relatives for each autopsy. The handling of the brain tissue and the diagnostic criteria have been described previously (42). Cerebrospinal fluid samples were collected from patients with Alzheimer’s disease.

### Real-time PCR

Total RNA from cultured cells was extracted using TRIzol (Invitrogen). One microgram of total RNA was reverse-transcribed using the SuperScript III First-Strand Synthesis System (Invitrogen) with random hexamers. The cDNAs were mixed with TaqMan Universal PCR master mix (Life Technologies) and amplified using an ABI PRISM 7900HT sequence detection system (Applied Biosystems). All primers and probes, *GALNT2* (Hs00189537_m1), *GALNT3* (Hs00237084_m1), *GALNT6* (Hs00926629_m1), and *18S rRNA* (Hs99999901_s1), were from Applied Biosystems. The levels of mRNA were normalized to the corresponding ribosomal RNA levels.

### Immunofluorescence

Cells metabolically labeled with Ac_4_GalNAz were fixed with 4% paraformaldehyde in PBS for 30 min, treated with 0.25% Triton X-100 for 15 min for permeabilizing the plasma membrane, and labeled with Alexa555-alkyne, in accordance with the manufacturer’s instructions (Invitrogen/Click-iT Cell Reaction Buffer Kit). For the analysis of Halo-APP770 uptake, the cells were incubated with non-permeable HaloTag Alexa 488 ligand (1 μM) or biotin ligand (25 μM), or permeable HaloTag TAMRA ligand (5 μM) for 5 to 30 min before fixation and permeabilizing the plasma membrane. For double staining, the cells were incubated at 4 °C with antibodies to the following: APP OX2 (1:75), adaptin-γ (1:100), EEA1 (1:100), Rab5 (1:150), Rab7 (1:100), GANLT2 (1:250), and DAPI. As antibodies for endogenous Rab11 were ineffective for immunocytochemistry, FLAG-tagged Rab11 was expressed in BMECs, and detected with anti-FLAG (M2, 1:100) to obtain a clear Rab11 signal. The next day, the cells were washed three times and incubated with the appropriate fluorescently labeled goat secondary antibodies (1:1000, Invitrogen) for 1 h at room temperature. Images were taken using an Olympus FV-1000 confocal microscope, with data acquisition and quantification of the signal or co-localization area being carried out using FV10-ASW ver.1.7 software (Olympus).

### Isolation of endothelial cells

Endothelial cells were isolated from mouse brains as previously reported (12, 43), except for the use of anti-CD146 antibody coupled with Dynabeads sheep anti-Rat IgG (Thermo Fisher Scientific).

### Western and lectin blot

The samples were subjected to SDS-PAGE (10% or 5%–20% gradient gel) and transferred to nitrocellulose membranes. For Western blot analyses, following incubation with 5% non-fat dried milk in TBS-containing 0.1% Tween-20, the membranes were incubated with anti-APP 22C11 (1:1000 dilution), anti-APP(C) (1:200–1000 dilution), anti-O-GlcNAc RL2 (1:500 dilution), or anti-actin (1:500 dilution) antibodies. Appropriate horseradish peroxidase-conjugated donkey anti-goat IgG (Jackson ImmunoResearch Laboratories) or anti-mouse and anti-rabbit IgG (GE Healthcare) antibodies were used as the secondary antibodies (1:1000 dilution). For the lectin pull-down experiment, the lysates (50 μg of protein) were incubated with 20 μl of *Arachis hypogaea* (PNA)-coupled agarose (J-oil Mills), and subjected to Western blot analysis with anti-APP(C). Signals were detected with SuperSignal West Dura Extended Duration Substrate (Thermo Fisher Scientific) using ImageQuant LAS-4000mini (GE Healthcare). The intensity of the resultant protein bands was quantified using ImageQuant TL software (GE Healthcare).

### Azide sugar labeling

BMECs were metabolically labeled with 100 μM Ac_4_GalNAz or Ac_4_ManNAz for 6 h. The Click-iT protein reaction buffer kit with Alexa555-labeled alkyne was used to label the fixed cells. For biochemical detection, the cell lysates (350 μg of protein) were incubated with TAMRA-conjugated DIBO for 3 h. sAPP in the medium was pulled down with heparin-agarose (Thermo Fisher Scientific Inc). The samples were then subjected to SDS-PAGE analysis. Fluorescent signals on the gel were visualized with Typhoon 9400 (GE Healthcare).

### Glycosidase treatment

Glycoproteins in the cell lysates (20 μg of protein) or medium that were pulled down with heparin-agarose (0.5 ml) were denatured and incubated with *Arthrobacter ureafaciens* sialidase (Nacalai Tesque, 4 milliunits) and/or O-glycosidase (New England BioLabs, 80,000 units), or peptide N-glycanase (PNGase, New England Biolabs, 1000 units) for 6 h. To remove O-GlcNAc, cell lysates (100 μg of protein) were incubated with O-GlcNAcase (5 μg, R&D Systems) at 37 °C for 2 h.

### Cell surface biotinylation assay

To measure the half-life of cell surface APP, or for biochemical analysis of cell surface biotinylated APP, Sulfo-NHS-LC-biotin was used from BMECs for 30 min at 4 °C. After washing the plates three times with 0.1 M glycine in PBS (pH 8.0) and once with PBS alone, cell lysates were prepared with TPER buffer (Thermo Scientific). For the internalization assay, BMECs were labeled with NHS-SS-biotin, and cultured for 0, 5, 10, or 60 min, after which cell surface biotin was removed using glutathione reagent as previously described (12, 44). Biotinylated proteins were pulled down with immobilized streptavidin. To check the incorporation of azide sugars in the biotinylated APP, following cell surface biotinylation with sulfo-NHS-LS-biotin, BMECs expressing APP770FLAG or control vector were metabolically labeled with azide sugars and lysed. Biotinylated proteins bound to immobilized streptavidin were eluted with 2 mM biotin, after which APP770FLAG was immunoprecipitated with anti-FLAG M2-agarose.

### Benzyl GalNAc treatment

Subconfluent BMECs were incubated in the presence of benzyl GalNAc (2 mM) for 18 h.

### *In vitro* GalNAcT assay

Human APP770-derived peptides, APP^347–358^ (QSLLKTTQEPLA), APP^361–372^ (PVKLPTTAASTP), and APP^646–657^ (ADRGLTTRPGSG), which were tagged with a 9-fluorenylmethyloxycarbonyl group at their N-terminus, were used as acceptor substrates. A series of recombinant soluble FLAG-GalNAcTs (45) was purified from the medium of overexpressing COS cells by immunoaffinity chromatography using M2 agarose. The concentration of each GalNAcT-FLAG enzyme was measured by performing immunoblot analysis together with standard FLAG-BAP fusion protein (Sigma-Aldrich) using the M2 antibody and adjusted. The standard enzyme reaction mixture containing 15 μM acceptor substrate, and the purified enzyme, 0.5 mM UDP-GalNAc in 25 mM Tris-HCl (pH 7.4), 5 mM MnCl_2_, and 0.1% Triton X-100 in a final volume of 20 μl, was incubated at 37 °C for 16 h, after which the reaction was terminated by boiling (30). The reaction products were purified with Millipore Ziptips and mixed with a matrix (2,5-dihydroxybenzoic acid), which was analyzed by Bruker Ultraflex MALDI mass spectrometry in the positive ion mode.

### Phylogenic analysis

Amino acid sequences of human ppGalNAc-T were obtained from the RefSeq database (46). Evolutionary analysis was conducted in MEGA6 (47) using the UPGMA method.

### Quantification of sAPP and Aβ

HeLa cells and BMECs were infected with adenovirus preparations for APP770-FLAG overexpression. HeLa cells and primary neurons were transfected with GalNAcT2-, T3-, or T6-encoding pcDNA using FuGENE6 and Lipofectamine 2000 (Invitrogen), respectively. BMECs were infected with adenovirus to express GalNAcT2, T3, or T6. To measure sAPP770, sAPP770β, Aβ40, and Aβ42 in the medium, a Human sAPP Total Assay Kit, an sAPPβ-w Assay Kit (highly sensitive) (IBL-Japan), and human amyloid β (1–40) and (1–42) assay kits were used, respectively. To measure O-GalNAc glycans on APP770, a 96-well plate coated with anti-OX2 antibody (IBL-Japan) was incubated with BMEC lysate for 16 h. *S. sieboldiana* agglutinin (SSA)-biotin (J-oil Mills, 1:1000) and streptavidin-HRP (1:1000) were then used for detection.

### Determination of O-glycosylation sites in APP770

HA-tagged human APP770 was expressed in HEK293T cells, and HA-sAPP770 purified from culture medium was precipitated in acetone at −30 °C for 16 h and then centrifuged at 12,000*g* for 10 min. The precipitated sample was reduced in 10 mM dithiothreitol (DTT) for 30 min at 56 °C, and alkylated with 20 mM iodoacetamide for 40 min at 25 °C in the dark. Then, the proteins were digested with 1.5 μg of Trypsin/Lys-C mix (Promega) for 16 h at 37 °C (800 rpm). The O-glycopeptides were precipitated with a five-fold volume of ice-cold acetone by centrifugation at 12,000*g* for 10 min (Fraction 1). The supernatant was collected in a separate tube and dried in a speed vac. concentrator. O-glycopeptide from the supernatant was enriched with GlycOCATCH (Genovis), in accordance with the manufacturer’s instructions. In brief, the supernatant reconstituted in 0.1% Triton x-100 in TBS was reacted with GlycOCATCH affinity resin at room temperature for 2 h with 10 units of SialEXO. The resin was washed three times with 0.5 M sodium chloride in TBS, and then eluted by incubating with 8 M urea for 5 min at room temperature with mixing (Fraction 2). Both fractions were combined, purified by GL-Tip SDB (GL Sciences), and then subjected to LC/MS. To identify the O-glycosylation site, a portion of the glycopeptide fraction was treated with OpeRATOR (Genovis) at 37 °C overnight.

The O-glycopeptides were separated on an EASY-nLC 1000 (Thermo Fisher Scientific) with an Acclaim PepMap100 C18 LC column (75 μm × 20 mm, 3 μm; Thermo Fisher Scientific) and a Nano HPLC Capillary Column (75 μm × 120 mm, 3 μm, C18; Nikkyo Technos). The eluents consisted of water containing 0.1% v/v formic acid (pump A) and acetonitrile containing 0.1% v/v formic acid (pump B). The O-glycopeptides were eluted at a flow rate of 0.3 μl/min with a linear gradient from 0% to 35% B over 40 min. Mass spectra were acquired on a Q Exactive mass spectrometer (Thermo Fisher Scientific) equipped with Nanospray Flex Ion Source (Thermo Fisher Scientific) operated in the positive ion mode. We used an Xcalibur 4.4 workstation (Thermo Fisher Scientific) for MS control and data acquisition. The spray voltage was set at 1.8 kV, while the capillary temperature was kept at 250 °C. The full mass spectra were acquired using an *m/z* range of 350 to 2000 with a resolution of 70,000. The product ion mass spectra were acquired against the ten most intense ions using a data-dependent acquisition method with a resolution of 17,500 and with normalized collision energy (NCE) of 27.

### Analysis of GalNAz-incorporated APP

FLAG-APP770 was expressed in BMECs using an adenoviral system and purified from cell lysates with anti-FLAG M2-agarose (Sigma-Aldrich). The lyophilized sample (30 μg of protein) was reduced with dithiothreitol (10 mg, 50 °C for 1 h) and alkylated with iodoacetamide (20 mg, room temperature for 30 min in the dark). After the reaction mixture had passed through a Nap-5 column (GE Healthcare) to remove excess dithiothreitol and iodoacetamide, the sample was digested with trypsin (2 μg, Promega) in 50 mM ammonium bicarbonate (100 μl) for 16 h at 37 °C. After boiling for 10 min, the sample was evaporated to dryness. The dried residue was dissolved with 12 μl of mobile phase (A), and a portion of it (5 μl) was used for LC-ESI MS and MS/MS analyses to determine the presence of glycopeptide containing GalNAz. The glycopeptide mixtures were separated using an ODS column (Develosil 300ODS-HG-5, 150 × 1.0 mm i.d.; Nomura Chemical). The mobile phases were (A) 0.08% formic acid and (B) 0.15% formic acid/80% acetonitrile. The column was eluted with solvent A for 5 min, at which point the concentration of solvent B was increased to 40% over 55 min at a flow rate of 50 μl/min using an Accela HPLC system (Thermo Fisher Scientific). The eluate was continuously introduced into an ESI source (LTQ Orbitrap XL; Thermo Fisher Scientific at the Natural Science Center for Basic Research and Development, Hiroshima University). MS and MS/MS spectra were obtained in the positive ion mode using Orbitrap (mass range: *m/z* 300–3000) and Iontrap (data-dependent scan of the top three peaks from a prepared list using CID), respectively. The voltage of the capillary source was set at 4.5 kV and the temperature of the transfer capillary was maintained at 300 °C. The capillary voltage and tube lens voltage were set at 15 V and 50 V, respectively.

## Data availability

This study includes no data deposited in external repositories.

## Supporting information

This article contains supporting information.

## Conflict of interest

The authors declare no competing financial interests.
