# Supplementary material for: O-GalNAc glycosylation determines intracellular trafficking of APP and Aβ production
Source: J Biol Chem. 2023 Jun 9;299(7):104905. doi: 10.1016/j.jbc.2023.104905 (PMC10344954; doi:10.1016/j.jbc.2023.104905)
Supplement: Supporting Figures S1–S5 [file mmc1.docx]

O-GalNAc glycosylation determines intracellular trafficking

of APP and Aβ production

**Yuriko Tachida^1,†^, Junko Iijima^2,†^, Kazuto Takahashi^2^, Hideaki Suzuki^2^, Yasuhiko Kizuka^3^, Yoshiki Yamaguchi^4^, Katsunori Tanaka^5,6^, Miyako Nakano^7^, Daisuke Takakura^8^, Nana Kawasaki^8^, Yuko Saito^9^, Hiroshi Manya^10^, Tamao Endo^10^, and Shinobu Kitazume^1,2,*^**

^1^Disease Glycomics Team, RIKEN, 2-1 Hirosawa, Wako 351-0198, Saitama, Japan, ^2^Department of Clinical Laboratory Sciences, School of Health Sciences, Fukushima Medical University School of Medicine, Fukushima 960-8516, Japan, ^3^Graduate School of Natural Science and Technology, Gifu University, Gifu, Japan; Institute for Glyco-core Research (iGCORE), Gifu University, Gifu, Japan, ^4^Division of Pharmaceutical Physical Chemistry, Tohoku Medical and Pharmaceutical University, Miyagi 981-8558, Japan, ^5^Department of Chemical Science and Engineering, School of Materials and Chemical Technology, Tokyo Institute of Technology, 2-12-1 Ookayama, Meguro-ku, Tokyo 152-8550, Japan, ^6^Biofunctional Synthetic Chemistry Laboratory, RIKEN Cluster for Pioneering Research, 2-1 Hirosawa, Wako, Saitama 351-0198, Japan,  ^7^Graduate School of Integrated Sciences for Life, Hiroshima University, Higashi-hiroshima 739-8530, Japan, ^8^Graduate School of Medical Life Science, Yokohama City University, Yokohama 230-0045 Japan, ^9^Department of Neuropathology, Tokyo Metropolitan Geriatric Hospital and Institute of Gerontology, Tokyo 173-0015, Japan, ^10^Molecular Glycobiology, Research Team for Mechanism of Aging, Tokyo Metropolitan Geriatric Hospital and Institute of Gerontology, Tokyo 173-0015, Japan.

Running title: Non-classical glycosylation of endothelial APP

**
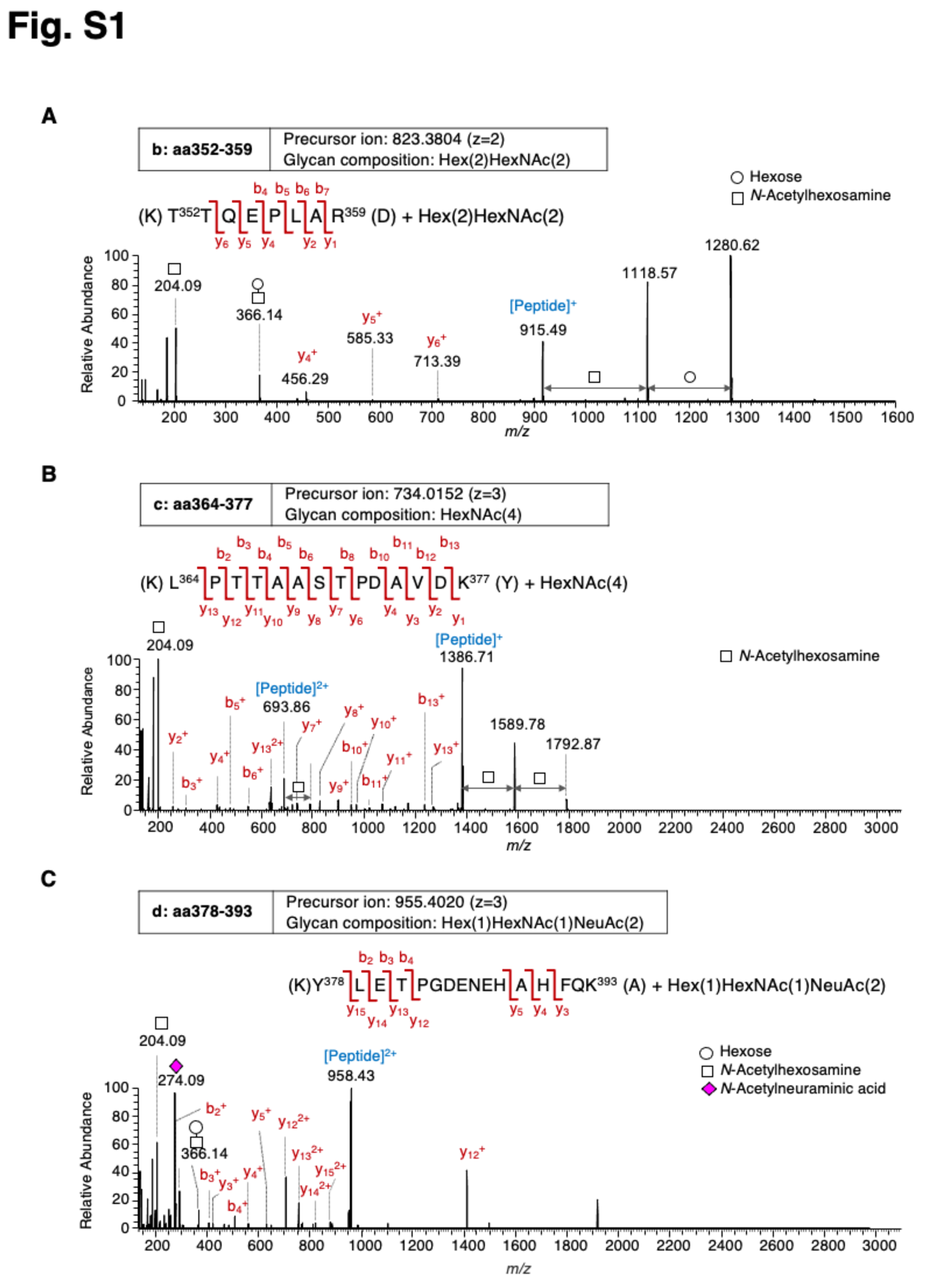
**

**
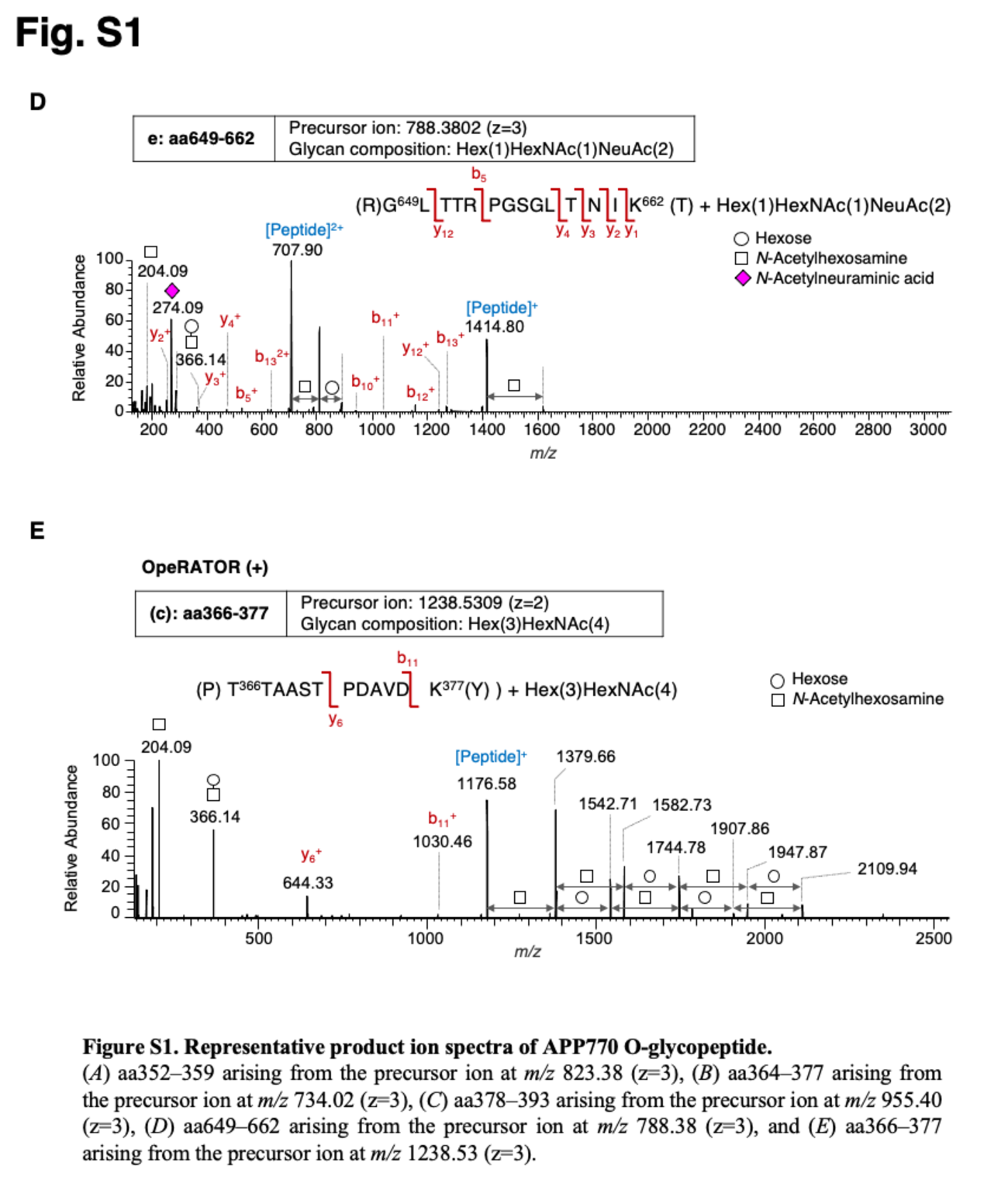
**

**Figure S1. Representative product ion spectra of APP770 O-glycopeptide.**

(*A*) aa352–359 arising from the precursor ion at *m/z* 823.38 (z=3), (*B*) aa364–377 arising from the precursor ion at *m/z* 734.02 (z=3), (*C*) aa378–393 arising from the precursor ion at *m/z* 955.40 (z=3), (*D*) aa649–662 arising from the precursor ion at *m/z* 788.38 (z=3), and (*E*) aa366–377 arising from the precursor ion at *m/z* 1238.53 (z=3).

**
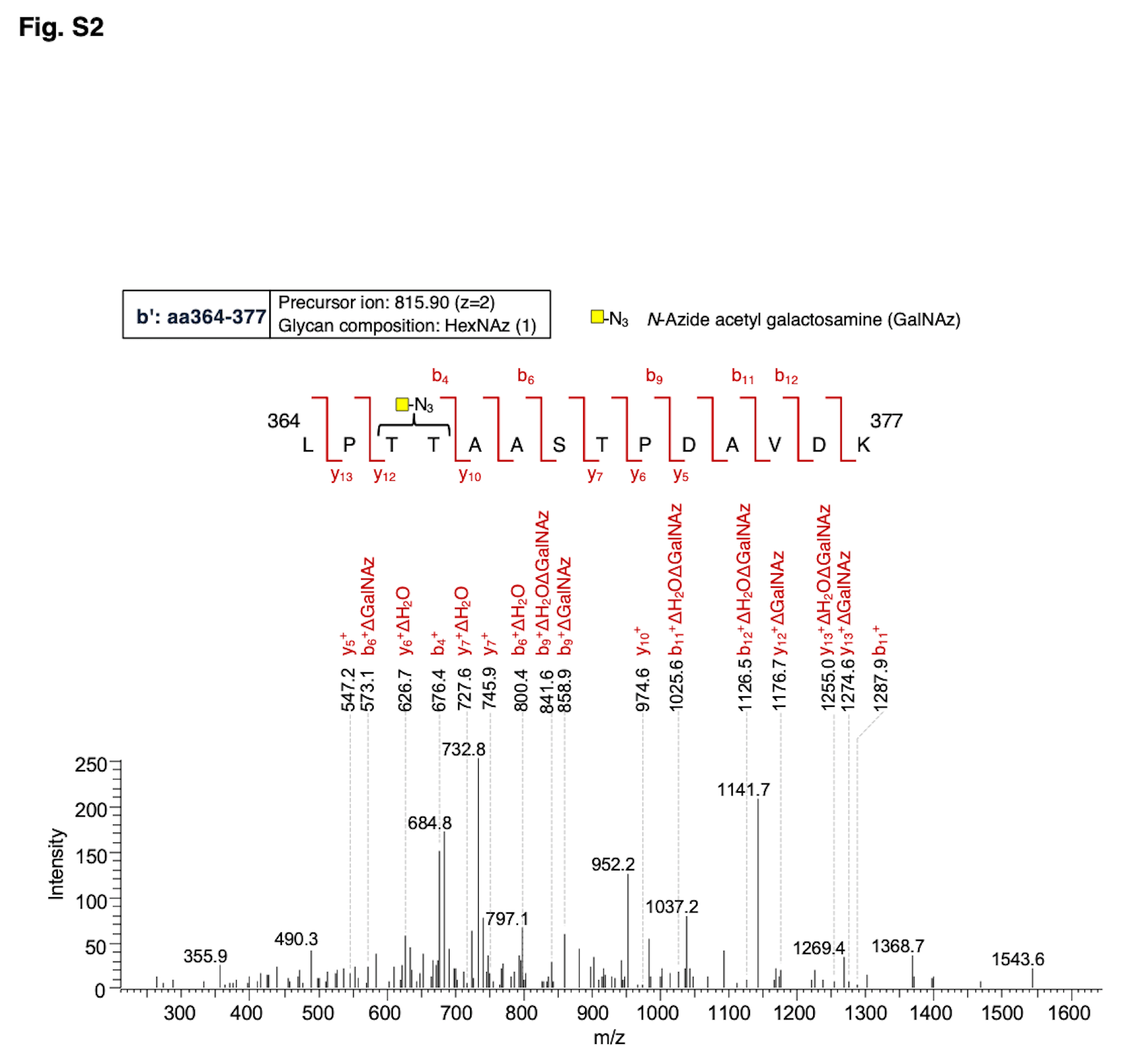
**

**Figure S2. Incorporation of GalNAz as part of O-glycans in APP770.** APP-FLAG, expressed using an adenovirus system in BMECs metabolically labeled with GalNAz, was immunopurified with an M2-coupled agarose column. The MS/MS of a precursor ion at *m/z* 815.90 representing LPTTAASTPDAVDK+HexNAz gives the indicative fragment b4 ion at *m/z* 676.4 representing LPTT+GalNAz and the y10 ion at *m/z* 974.6 representing AASTPDAVDK.

**Figure S3. Metabolic labeling with GalNAz results in nuclear azide signals derived from O-GlcNAc signals.**

IdlD cells, which lack UDP-galactose epimerase (GALE) activity, and their parental CHO cells were incubated with GalNAz. After 6 or 24 h of incubation, the cells were fixed, reacted with Alexa555-alkyne and DAPI, and analyzed by immunofluorescence microscopy. Scale bar, 20 μm.


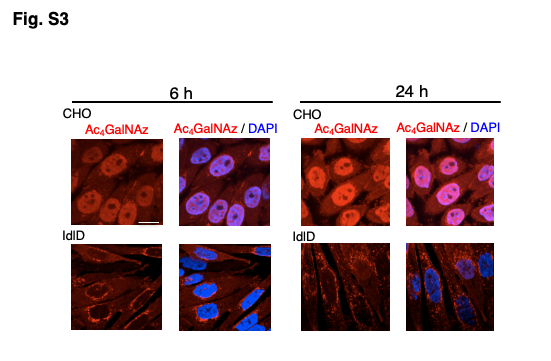


**Figure S4.** **O-GalNAz glycans are enriched in the recycling endosome.**

(*A*) Immunostaining of BMECs with APP-FLAG and several organelle markers (adaptin-γ, EEA1, Rab5, Rab7, and Rab11). Scale bar, 20 μm (and 4 μm for magnified image). (*B*) Quantitative analysis of the percentage of APP and organelle marker co-localization is shown as mean ± SEM. (n=4) in (*A*). (*C*) Immunostaining of BMECs for O-GalNAz glycans and several organelle markers. Scale bar, 20 μm (and 4 μm for magnified image). (*D*) Quantitative analysis of the percentage of O-GalNAz glycan and organelle marker co-localization is shown as mean ± SEM (n=4) in (*C*).
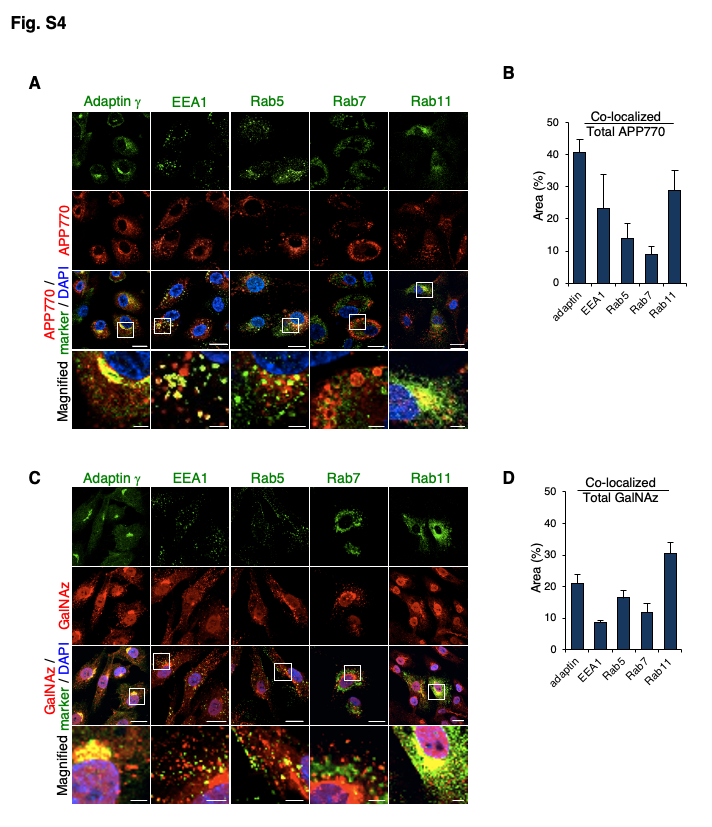


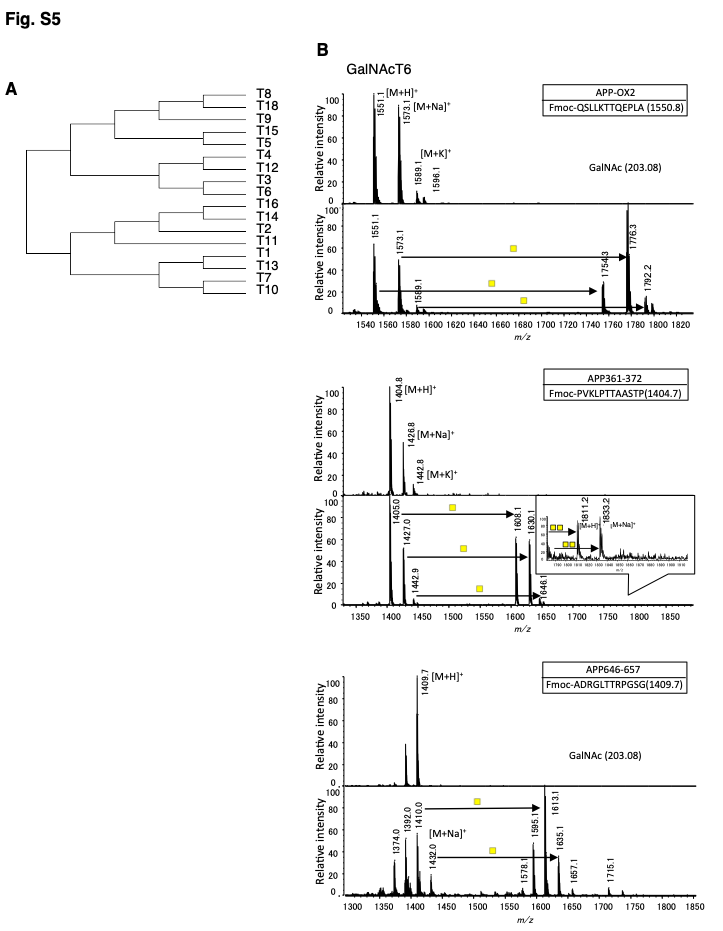


**Figure S5. *In vitro* GalNAc-T assay shows the incorporation of GalNAc into APP-derived peptide.**

(*A*) Phylogenic analysis of the human GalNAc-T family. (*B*) Soluble recombinant GalNAc-T6 was incubated with three kinds of APP-derived peptide and UDP-GalNAc. The reaction products were partially purified by Millipore Ziptips and analyzed by mass spectrometry (AXIMA-QIT TOF-MS, Shimadzu Biotech). MS spectra of the reaction products (lower) show the incorporation of one or two GalNAc residues into the peptide substrate (upper).
